# An Endogenous Electron Spin Resonance (ESR) Signal Discriminates Nevi from Melanomas in Human Specimens: A Step Forward in Its Diagnostic Application

**DOI:** 10.1371/journal.pone.0048849

**Published:** 2012-11-07

**Authors:** Eleonora Cesareo, Liudmila Korkina, Gerardino D’Errico, Giuseppe Vitiello, Maria Simona Aguzzi, Francesca Passarelli, Jens Z. Pedersen, Antonio Facchiano

**Affiliations:** 1 Laboratory of Tissue Engineering & Skin Pathophysiology, Istituto Dermopatico dell’Immacolata IDI-IRCCS, Rome, Italy; 2 Department of Chemical Sciences, University of Naples “Federico II”, Naples, Italy; 3 Consorzio per lo Sviluppo dei Sistemi a Grande Interfase (CSGI), Florence, Italy; 4 Laboratory of Vascular Pathology, Istituto Dermopatico dell’Immacolata (IDI-IRCCS), Rome, Italy; 5 Histopathology Service, Istituto Dermopatico dell'Immacolata (IDI-IRCCS), Rome, Italy; 6 Department of Biology, University of Tor Vergata, Rome, Italy; University of Connecticut Health Center, United States of America

## Abstract

Given the specific melanin-associated paramagnetic features, the Electron Spin Resonance (ESR, called also Electron Paramagnetic Resonance, EPR) analysis has been proposed as a potential tool for non-invasive melanoma diagnosis. However, studies comparing human melanoma tissues to the most appropriate physiological counterpart (nevi) have not been performed, and ESR direct correlation with melanoma clinical features has never been investigated. ESR spectrum was obtained from melanoma and non-melanoma cell-cultures as well as mouse melanoma and non-melanoma tissues and an endogenous ESR signal (g = 2.005) was found in human melanoma cells and in primary melanoma tissues explanted from mice, while it was always absent in non-melanoma samples. These characteristics of the measured ESR signal strongly suggested its connection with melanin. Quantitative analyses were then performed on paraffin-embedded human melanoma and nevus sections, and validated on an independent larger validation set, for a total of 112 sections (52 melanomas, 60 nevi). The ESR signal was significantly higher in melanomas (p = 0.0002) and was significantly different between “Low Breslow’s and “High Breslow’s” depth melanomas (p<0.0001). A direct correlation between ESR signal and Breslow’s depth, expressed in millimetres, was found (R = 0.57; p<0.0001). The eu/pheomelanin ratio was found to be significantly different in melanomas “Low Breslow’s” vs melanomas “High Breslow’s” depth and in nevi vs melanomas “High Breslow’s depth”. Finally, ROC analysis using ESR data discriminated melanomas sections from nevi sections with up to 90% accuracy and p<0.0002. In the present study we report for the first time that ESR signal in human paraffin-embedded nevi is significantly lower than signal in human melanomas suggesting that spectrum variations may be related to qualitative melanin differences specifically occurring in melanoma cells. We therefore conclude that this ESR signal may represent a reliable marker for melanoma diagnosis in human histological sections.

## Introduction

Skin melanoma is one of the most aggressive tumours in humans, showing high mortality at the metastatic stage, and increasing incidence worldwide. [1] Melanoma accounts for about 4% of skin cancers, causing however about 80% of skin cancer-related deaths in western countries. Despite promising recent improvement at the therapeutic level, [2]–[6] surgical excision remains at this moment the most effective treatment at early stages, while therapeutic interventions have weak efficacy at advanced phases due to high metastatic potential and resistance to currently available therapies. [7].

Improving early diagnosis may therefore strongly affect melanoma-related mortality. Melanoma diagnosis routinely starts from non-invasive dermatoscopy- and epiluminescence-based skin inspection, to identify phenotypic features of the pigmented lesion. [8] Trained dermatologists still experience significant error-rate giving misdiagnosis and delay in treatment, and formal diagnosis still requires to be confirmed by histological analysis. [9] Hence, alternative non-invasive procedures are needed to improve the early non-invasive diagnostic accuracy.

Several reports indicate melanogenesis as a key process in the melanoma biology. [10], [11] Melanin synthesis involves an oxidation/reduction reactions chain leading to the synthesis of final organic polymers. The intermediate free radicals formed within such process [12] give melanin paramagnetic properties. [13] Besides free radicals, melanin may also contain or interact with metal ions and paramagnetic gases (dioxygen, nitric oxide) which also contribute to its paramagnetic properties. [14] ESR spectroscopy is the technique of choice to detect and to investigate free radicals. As such, it has long been used in melanin basic research since melanin ESR signal is stable, [15]–[17] resistant to chemical degradation, [18] and different in eumelanin from pheomelanin. [19], [20].

Previous studies investigated ESR spectra of melanoma tissues under different conditions, [21]–[29] including formaline fixed-, or frozen-, or paraffin-embedded specimens. However, a large study investigating ESR spectra in human melanoma specimens compared to human nevus specimens is still lacking at this moment.

The main goal of the present study was to provide strong support to the use of ESR spectroscopy as a reliable diagnostic help in melanoma management. To this aim we identified an endogenous ESR signal (g = 2.005) in melanoma and non-melanoma human cell lines, then investigated this signal in mouse melanoma tissues. Finally, we investigated ESR signal in human melanoma specimens compared to human nevus specimens. A specific ESR signal was found in melanoma human tissues, significantly different from the one recorded in nevus paraffin-embedded specimens; ROC analysis showed that ESR signal is able to discriminate human melanoma sections from nevi, with very high accuracy.

## Methods

### Cell Cultures

Five human melanoma cell lines from both primary and metastatic melanomas were purchased from the American Type Culture Collection (ATCC, Manassas, VA) and cultured according to the manufacturer's instructions. SKMEL-28 (ATCC number HTB-72), SKMEL-2 (ATCC number HTB-68) and amelanotic C32 (ATCC number CRL-1585) cell lines were grown in Eagle’s Minimum Essential Medium (EMEM) with FBS to a final concentration of 10%. SKMEL-31 cell line (ATCC number HTB-73) was grown in EMEM with FBS to a final concentration of 15%; SKMEL-3 cell line (ATCC number HTB-69) was grown in McCoy’s 5a medium with FBS to a final concentration of 15%. SKMEL-110 human metastatic melanoma cells [4] were a kind gift of Dr. Cirielli (IDI-IRRCS, Rome) and were grown in Dulbecco's modified Eagle's medium (DMEM) with FCS to a final concentration of 10%. NHEM-neo primary melanocytes (Cambrex) were grown in MBM-2 supplemented with MGM-4 SingleQuots (CaCl2, FGF-2, PMA, rh-Insulin, Hydrocortisone, bovine pituitary extract (BPE), FBS and Gentamicin/Amphotericin) (Cambrex, Charles city). Normal Human Umbilical Vein Endothelial Cells (HUVEC) were from Lonza Inc. **(**Walkersville, MD) and were grown in Endothelial Cell Basal Medium-2 (Clonetics/BioWhittaker Inc., Charlotte, NC) supplemented with: Hydrocortisone, hFGF-2, VEGF, R3-IGF-1, Ascorbic Acid, Heparin, FBS, hEGF, GA-1000 (Clonetics/BioWhittaker, Inc., Charlotte, NC).

Human adult low Calcium Temperature keratinocytes (HaCaT) were a kind gift of Dr. Pastore (IDI-IRRCS, Rome) [30] and were grown in DMEM medium with FBS 10% final concentration.

Cells were grown in 75 cm^2^ flasks and media was changed every other day. Once a 75% confluence was reached, cells were trypsinized, harvested by centrifugation (10 minutes at 1500 RPM), washed and transferred into glass flat capillaries. ESR analysis was performed on intact cells; viability assay by trypan blue exclusion test always indicated at least 98% of live cells. Commercially available melanoma cells were used at maximum fifth passage, unless differently specified.

### *In vivo* Mouse Melanoma Model

For *in vivo* mouse experiments, murine B16F10 melanoma cells were purchased from American Type Culture Collection (ATCC, Manassas, VA) (ATCC number CRL-6475) and grown in DMEM (Hyclone, Logan, UT) with 10% FCS (Hyclone, Logan, UT) [5]. Media were completed by the addition of glutamine (2 mM) and penicillin/streptomycin (50 U/ml- 50 µg/ml) (Gibco, Carlsbad, CA). Cells were grown at 37°C with 5% CO_2_ and subsequently injected in the dorsal skin of 20 weeks-old male C57BL/6 mice (number of animals = 5) according to a previously reported procedure [4]. Primary melanomas were removed 2 weeks after cell-inoculation and kept on ice in a PBS solution until ESR analysis.

### Human Paraffin-embedded Tissue Sections

112 human paraffin embedded melanoma and nevus specimens were prepared as previously described [31], [32]. Two experimental sets were analyzed: 26 paraffin-embedded specimens (40 microns slides) of human nevus or melanoma (13 nevi and 13 melanomas) were assigned to the “Measuring set” and were analyzed first. Then a second independent set of 86 paraffin-embedded specimens (47 nevi and 39 melanomas) was assigned to the “Validation set” and analyzed. ESR measurements were carried out according to the methodology reported below. Paraffin embedded slices were weighed and ESR data were all normalized accordingly. Slice of pure paraffin revealed no ESR signal.

### Ethics Statement

The data were collected within a study approved by the local Ethic Committee (IDI IRCCS and San Carlo Hospital Ethical Committee Protocol, July 19th 2005; Reg. N. 154); written informed consent was obtained and all data were analyzed anonymously.

### ESR Analysis

For ESR spectra on intact cells, signals were recorded using 80-µl samples in flat glass capillaries (inner dimensions 0.4×4.0 mm) to optimize instrument sensitivity as previously described for cells in aqueous suspension. [33] Measurements on cells were performed at room temperature with an ESP300 X-band instrument (Bruker, Karlsruhe, Germany) equipped with a high sensitivity TM_110_-mode cavity. Spectra were measured over a 200 G range using 20 mW power, 2.0 G modulation, and a scan time of 42 s; 4 single scans were accumulated to improve the signal-to-noise ratio. Qualitative measurements of tissues and human paraffin-embedded sections were performed at room temperature in circular glass capillaries (inner diameter 1.10 mm) using the apparatus and experimental settings described above. Twenty four single scans were accumulated to improve the signal-to-noise ratio.

Quantitative measurements of the samples belonging to the “Measuring set” and “Validation set” were carried out on a different instrument (Bruker Elexys E500 X-band, equipped with a super-high sensitivity probe head) [34], [35]. Such measures were carried out over a 100 G range using 20 mW power, 3.0 G modulation, and a scan time of 42 s; 64 single scans were accumulated to improve the signal-to-noise ratio. The amplitude of the field modulation was preventively checked to be low enough to avoid detectable signal overmodulation.

The other experimental parameters have been set as follow: conversion time : 83.69 ms, time constant :163.84 ms, receiver gain 60 dB, number of points:1024. For selected samples signal saturation was checked to be reached above 60 mW microwave power.

The g value has been evaluated by means of an internal standard (DPPH). In details, DPPH was inserted in a very thin capillary. In turn, this capillary was inserted in the measuring test tube co-axially with the investigated samples.

ESR quantitative data were expressed both as peak-to-peak amplitude and as double integral intensity; linewidth of all samples was also measured.

In each sample of paraffin embedded samples, the ratio between the height of the major peak (a) and the height of a weak shoulder at lower field (g ≈ 2.01) (b) has been measured. This ratio is reported to correlate in a linear manner with the proportion between eumelanin and pheomelanin monomers in a copolymer [18], [20].

### Statistical Analysis

For statistical analysis, the entire set of paraffin-embedded samples was divided in groups and subgroups, according to different parameters (diagnosis, sex, body location of lesions, Breslow’s depth) (Table 1). The statistical analyses were performed using the Graph-Pad Prism 5 software; D’Agostino and Pearson normality Test was performed and groups showing normal distribution were analyzed with T test, while groups showing not-normal distribution were analyzed by Mann-Whitney Test; two-tailed p<0.05 was chosen as significance threshold.

**Table 1:** Nevi and melanomas subgroups used for statistical evaluation; numerosity of each subgroup is reported.

	TOTAL	TRUNK	LIMBS	HEAD and NECK	MALE	FEMALE
**NEVI**	**60**	**38**	**14**	**8**	**33**	**27**
**MELANOMA**	**52**	**24**	**18**	**9**	**25**	**27**
**MELANOMA LOW BRESLOW'S DEPTH** **(<1 mm)**	**19**	**8**	**6**	**5**	**12**	**7**
**MELANOMA HIGH BRESLOW'S DEPTH (≥1 mm)**	**33**	**16**	**12**	**4**	**13**	**20**

ANOVA analysis was carried out with the “Bonferroni Multiple Comparison Test”; two-tailed p<0.01 was chosen as significance threshold. Spearman correlation was also performed to compute ESR signal amplitude correlation with Breslow’s depth (expressed in mm) in all melanoma samples.

ROC analysis was also carried out to measure the ability to discriminate nevi from melanoma subgroups.

## Results

### ESR Spectra in Melanoma Cell Cultures

**Figure 1:**
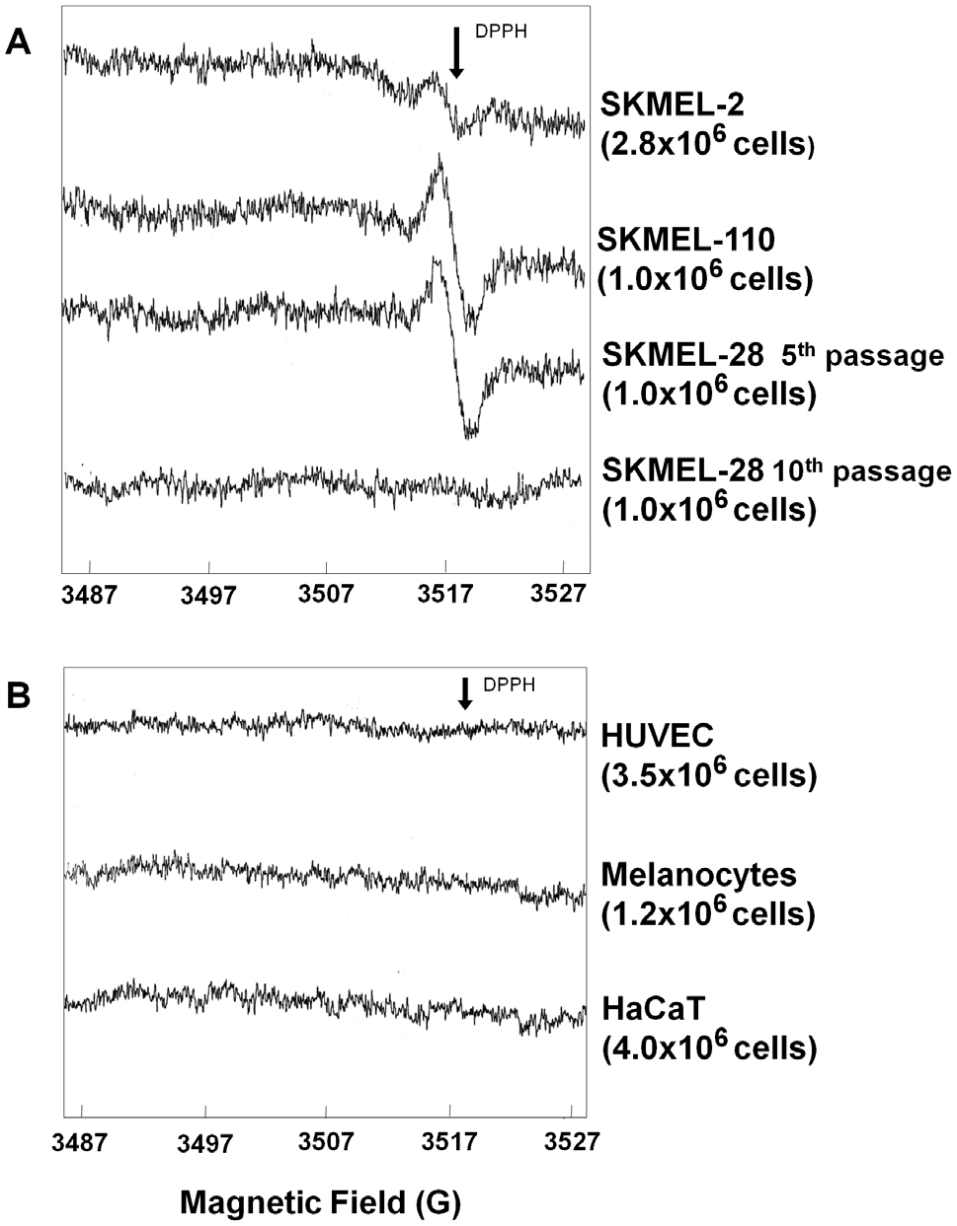
ESR spectra recorded in different melanoma and non-melanoma cell lines. **A)** Melanoma cell-lines showing the ESR signal. The signal observed in SKMEL-28 melanoma cell line at passage 5th was lost at passage 10th. **B)** Control cells lines (i.e. non- melanoma cell lines) showing no ESR signal. DPPH arrow indicates the position of the standard free radical signal (1, 1-diphenyl-2-picrylhydrazyl).

Six human melanoma cell lines from primary and metastatic melanomas were analyzed. Three melanoma cell lines (SKMEL-110, SKMEL-28 and SKMEL-2) showed an ESR signal (g = 2.005) (Fig. 1A). Spectra obtained from SKMEL-110 and SKMEL-28 were stable and intense, by repeated measure of the ESR signal at hours of distance. On the contrary, spectra from SKMEL-2 showed a faint peak, which disappeared when the measure was repeated after one hour (data not shown). No signal was detected in three other melanoma cell lines analyzed (namely, SKMEL-3, SKMEL-31 and C32) (spectra not shown). Fig. 1A indicates that SKMEL-28 cell-line shows a remarkable ESR signal at the 5^th^ culture passage, while the same signal is lost at the 10^th^ culture passage.

Human endothelial cells (HUVEC), human keratinocytes (HaCaT) and human primary melanocytes were used as controls and did not show the ESR signal found in melanoma cells (Fig. 1B).

### ESR Spectra in Fresh Samples of Primary Mouse Melanomas and Healthy Tissues

**Figure 2:**
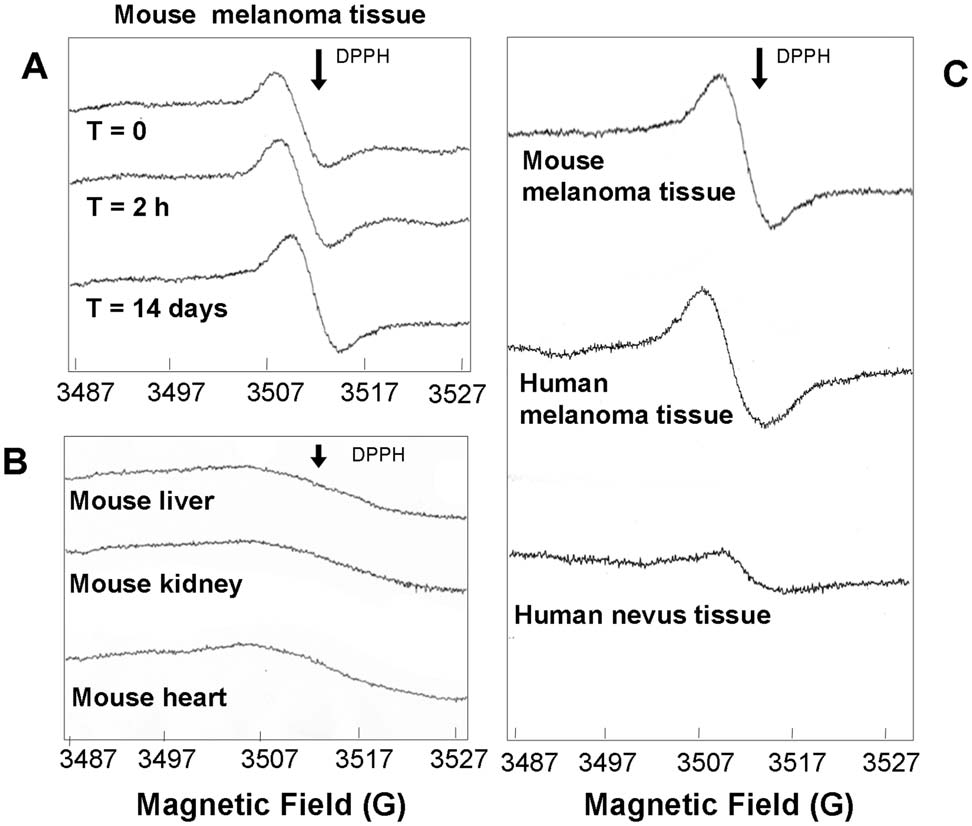
ESR spectra of murine- and human- melanoma and healthy tissues. **A)** Murine B16F10 melanoma cells were injected in 5 mice in order to produce primary melanomas. Mice were sacrificed 14 days after the cell injection and tumours were collected for ESR analysis. The spectra show the presence of a strong signal located at the same position as observed in human melanoma cells. Signal was stable over time (recorded after 2 hours and after 14 days upon frozen storage). **B)** Murine tissues from liver, kidney and heart do not show ESR signal in the same magnetic field range. **C)** ESR spectra of formalin-fixed paraffin-embedded sections of human melanoma, human nevus tissue and fresh mouse melanoma tissue. DPPH arrow indicates the position of the standard free radical signal (1, 1-diphenyl-2-picrylhydrazyl).

Freshly excised primary mouse melanomas were then collected from 5 different mice, previously inoculated subcutaneously with B16F10 cells (according to previously published protocol) [4]. ESR scanning was then carried out onto such samples under identical spectral conditions as reported for cultured cells. The analysis confirmed the presence of a strong ESR signal matching the one observed in melanoma cell lines. The signal was intense and stable when measured again at room temperature after 14 days of sample storage at −80°C (Fig. 2A). Liver, kidney and heart tissues taken from the same animals were used as controls, and a weak and broad ESR signal was recorded, different from the sharp signal found in mouse melanomas (Fig. 2B **)**.

### ESR Spectra in Paraffin-embedded Sections of Human Melanomas and Human Nevi

ESR spectra were then collected in human melanoma paraffin–embedded specimens and in human nevus paraffin-embedded specimens (40 microns each), in order to perform more quantitative analyses and verify the hypothesis that ESR may help discriminate melanoma specimens from healthy controls.

A preliminary qualitative analysis of paraffin-embedded nevi and melanomas indicated that an ESR signal is present in human specimens, corresponding to the ESR signal observed in mouse melanoma tissues and that the signal is lower in nevi than in melanomas (Fig. 2C).

A quantitative analysis was then carried out on a group of 26 formalin-fixed paraffin-embedded blocks of human skin melanomas and nevi; this samples-group was named “Measuring Set”.

**Figure 3:**
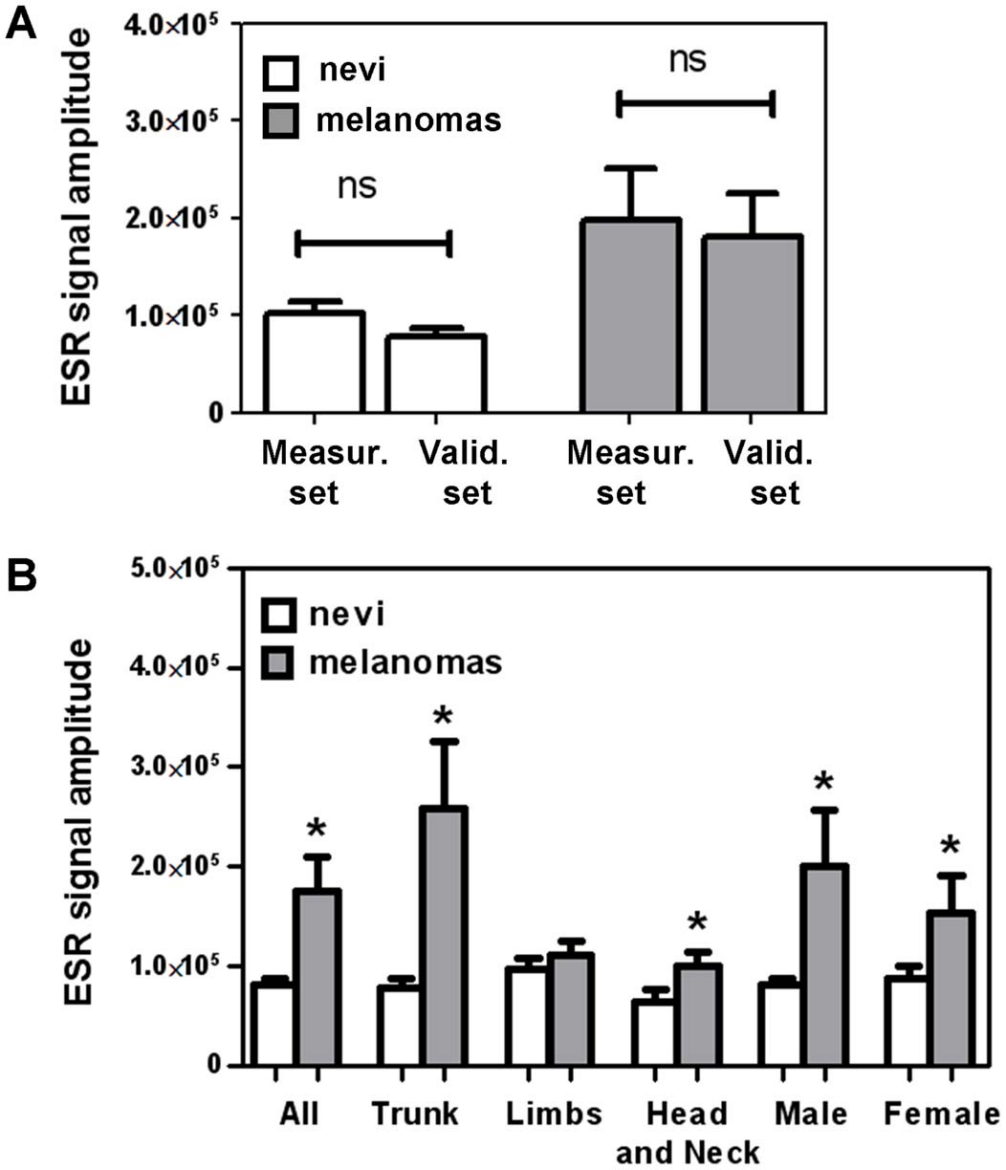
ESR signal of nevi and melanomas groups and subgroups. Bars report the ESR mean value of each subgroup with SEM. **A)** ESR value recorded in nevi (white bars) and melanomas (grey bars) in the “Measuring Set” and “Validation Set”. **B)** Comparison of nevi (white bars) vs melanoma (grey bars) in each subgroup of the “All Set”; * indicates p≤0.05 for “Female” and “Head and Neck”, in all other cases p<0.01; *ns* stands for “not significant”.

To validate such analysis, an independent larger samples set (named “Validation set”) of human melanomas and nevi was investigated (N = 86) using the same instrument and the same set up. Results shown in Fig. 3A (reported as mean ± SEM) indicate similar data in the two sets, namely they indicate that nevi of the “Measuring set” show no significant difference vs nevi of the “Validation set”, and melanomas of the “Measuring set” show no significant difference vs melanomas of the “Validation set”, allowing us to conclude that two independent samples sets are not significantly different. Further, in either sets melanomas show a signal higher than nevi.

Therefore the two sets were combined in one all-inclusive set (named “All set”) to achieve enough sample numerosity and to further analyze demographic and clinical features such as sex, body-location and tumour thickness (Table 1).

Nevus and melanoma samples of the “All set” were divided in subgroups according to sex and lesion body location (“Trunk”, “Limbs” and “Head and Neck”). Mann-Whitney Test revealed that in all subgroups (except “Limbs” location) a significantly different signal was found between nevi and melanomas (p≤0.05). The superimposition of the selected peak of 8 nevi and 8 melanomas is reported in Figure S1.

**Figure 4:**
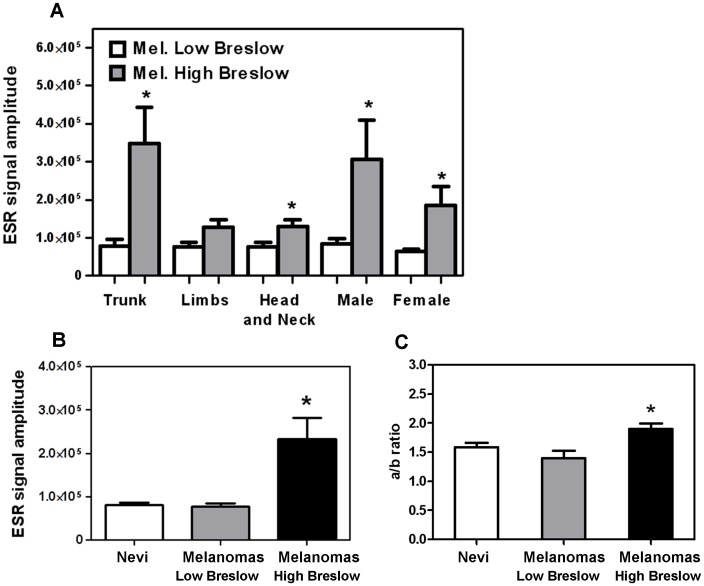
ESR signal within melanoma subgroups. **A)** Each subgroup was classified according to tumour thickness (High or Low Breslow’s depth). Bars report the ESR mean value of each subgroup with SEM; * indicates p≤0.05. **B)** ANOVA analysis with Bonferroni Multiple Comparison Test, within the group containing nevi, melanomas “Low Breslow’s depth” (<1 mm) and melanomas “High Breslow’s depth” (≥1 mm); * indicates p<0.01 of melanomas “High Breslow” vs nevi and vs melanomas “Low Breslow”). **C)** ANOVA analysis with Bonferroni Multiple Comparison Test of the eu/pheomelanin ratio (a/b), of nevi, melanomas “Low Breslow’s depth” (<1 mm) and melanomas “High Breslow’s depth” (≥1 mm) groups; * indicates p<0.01 of melanomas “High Breslow” vs nevi and vs melanomas “Low Breslow”).

Additional statistical analyses were carried out within melanomas subgroups. Each subgroup was classified according to tumour thickness, (“High” or “Low” Breslow’s depth) (Table 1), i.e. a parameter strongly related to the prognosis, being “High Breslow” associated to a worse prognosis. The ESR signal was significantly higher in samples with “High Breslow” in all melanomas subgroups (p<0.05) except “Limbs” (Fig. 4A).

An additional ANOVA analysis confirmed the highly significant difference of the melanomas ESR signal with “High Breslow’s depth” *vs* nevi and melanomas “Low Breslow” (Fig. 4B).

**Figure 5:**
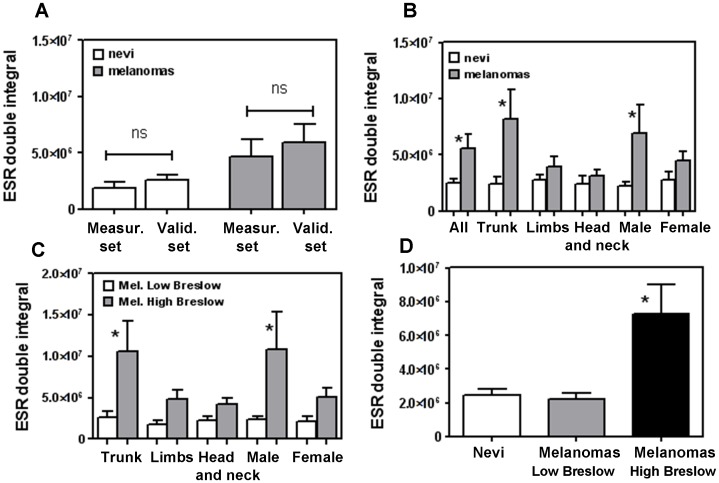
ESR double integral values. Calculations reported in Fig. 3 and Fig. 4 carried out on double integral values. A) ESR value recorded in nevi (white bars) and melanomas (grey bars) in the “Measuring Set” and “Validation Set”; *ns* stands for “not significant”. **B)** Comparison of nevi (white bars) vs melanoma (grey bars) in each subgroup of the “All Set”; * indicates p<0.01. **C)** Each subgroup was classified according to tumour thickness (High or Low Breslow’s depth). Bars report the ESR mean value of each subgroup with SEM; * indicates p≤0.05. **D)** ANOVA analysis with Bonferroni Multiple Comparison Test of nevi, melanomas “Low Breslow’s depth” (<1 mm) and melanomas “High Breslow’s depth” (≥1 mm) groups; * indicates p<0.01.

All calculations reported in Fig. 3 and Fig. 4 were carried out on amplitudes values; each calculation has also been performed on double-integral values reaching almost superimposable results as compared to amplitudes (Fig. 5).

A correlation analysis by Spearman Test carried out in the 52 melanoma samples indicated a strongly significant correlation (R = 0.57; p<0.0001) between ESR signal amplitude and the corresponding Breslow’s depth value expressed in millimetres. Similar results were observed using integral values (R = 0.42; p = 0.002).

The variation of the eumelanin/pheomelanin ratio (a/b) (see methods) was also investigated indicating a significant difference of melanomas “Low Breslow” vs “High Breslow” melanomas (p<0.004) and nevi vs “High Breslow” melanomas. (p<0.009) ANOVA analysis carried out on a/b ratio confirmed a significant difference (Fig. 4C).

**Figure 6:**
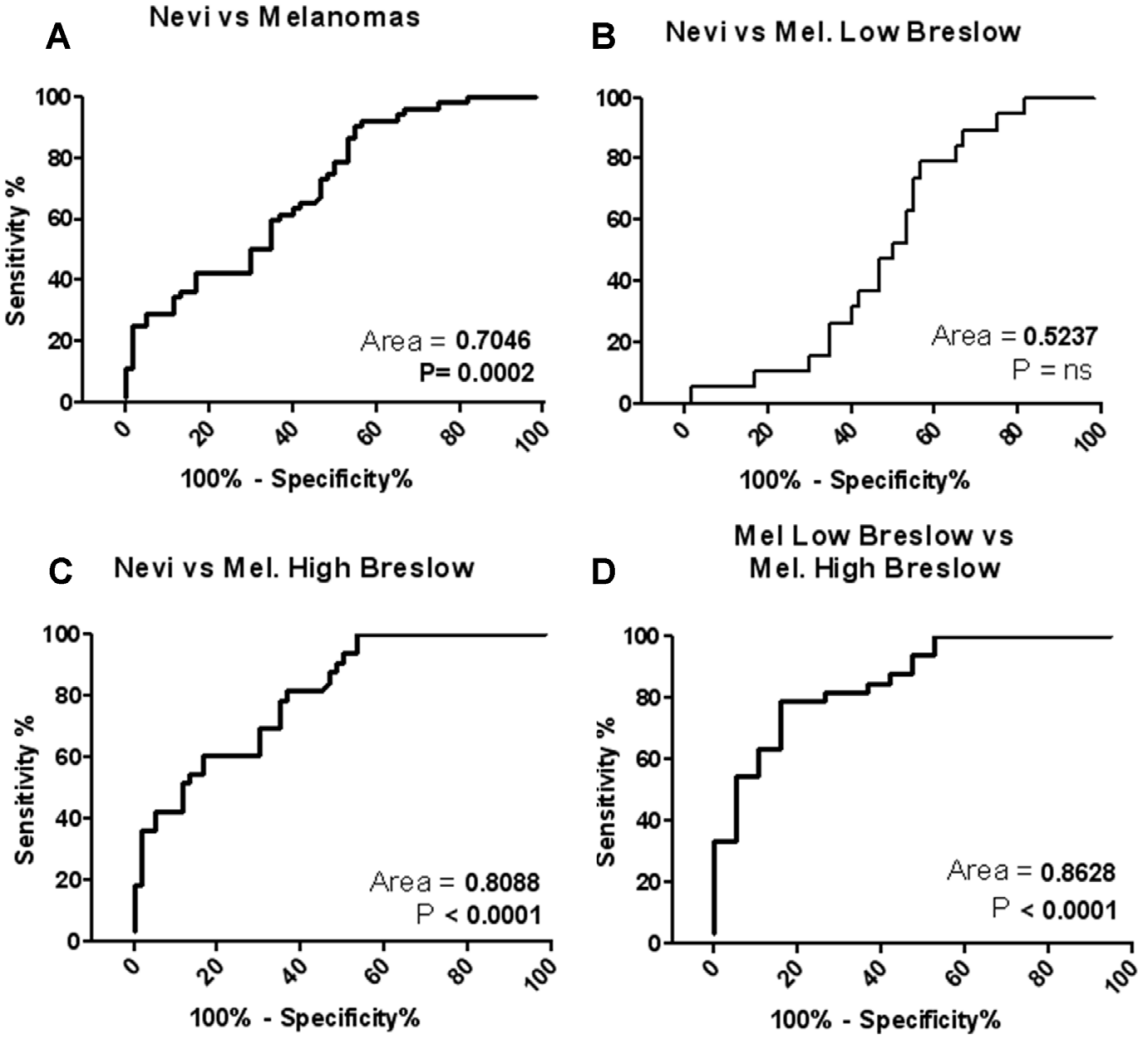
ROC analysis. **A)** Nevi vs Melanomas; **B)** Nevi vs Melanomas “Low Breslow”; **C)** Nevi vs Melanomas “High Breslow”; **D)** Melanomas “Low Breslow” vs Melanomas “High Breslow”; *ns* stands for “not significant”.

ROC analysis was then carried out to test the ability of ESR signal to discriminate nevi from melanomas in paraffin-embedded sections. The computed area under the ROC curve quantifies the ability to discriminate controls from melanoma patients taking into account both sensitivity and specificity. A value of 1 indicates the ability to discriminate 100% of patients from controls and corresponds to a curve mostly left-shifted in the graph. According to such analysis, nevi were effectively discriminated from melanomas, showing a ROC area of 0.70 (corresponding to 70% accuracy; p<0.0001; Fig. 6A). Nevi were also effectively discriminated from melanomas “High Breslow’s depth”, with a ROC area of 0.81 (corresponding to 81% accuracy; p<0.0001; Fig. 6C). Melanomas “Low Breslow” were effectively discriminated from melanomas “High Breslow” with a ROC area of 0.86 (corresponding to 86% accuracy; p<0.0001; Fig. 6D). No significant discrimination was detected between nevi and melanomas “Low Breslow” (Fig. 6B).

These data were achieved using amplitude values, which have a low coefficient variation. When double integral values were used for ROC analyses, the ROC area was lower, while the statistical significance remained very high (p<0.005 in all cases) (Figure S2).

### Linewidth Measure

It has been reported that peak-to-peak amplitude is an indicative quantitative measure of the ESR spectra as effective as the corresponding double integral, provided linewidth remains constant. We therefore measured the linewidth of ESR peaks in all samples of the “All set”; the values measured in nevi were much similar to melanomas (8.0 G vs 7.8 G, p = 0.52) as well as in melanomas “High” vs melanomas “Low” Breslow (7.8 G vs 7.9 G, p = 0.4); the entire nevi/melanomas population showed a mean linewidth of 7.9 G ±0.15 SEM.

## Discussion

Significant advances have been recently achieved on molecular melanoma markers [36]–[38]; however, clinical application to non-invasive melanoma diagnosis is still far to come. Early diagnosis is a key request in aggressive tumours such as melanoma; currently, trained dermatologists perform visual- and epiluminescence-based macroscopical inspection, and suspect melanoma is then confirmed by histological examination. ESR-based *in situ* analysis of pigmented skin lesions has been suggested as a possible non-invasive diagnostic tool, according to results obtained on melanoma cell lines and melanoma tissues [29], [39]–[41] and in animal models of melanoma [39], [42]. As possible melanoma markers, stable melanin granule-connected [29] or cellular membrane-associated [43] or UV-induced short living melanin- and oxygen-derived ESR signals have been proposed [44], [45]. However, a statistical analysis of endogenous ESR signal on human melanomas and nevi samples collection has never been performed.

In the present study different melanoma cell lines are reported to exhibit endogenous ESR signal at g = 2.005±0.001 in the spectrum obtained under specific physical settings while other melanoma cell lines do not present the ESR signal.

Such difference may be related to heterogenic features (e.g. primary or metastatic) and growth related properties (e.g. doubling time) of cultured melanoma cell lines, reflecting the clinical variability observed in melanoma patients [46]. Noteworthy in our experimental in *vitro* conditions the signal recorded in SKMEL-28 at the 5^th^ culture passage was lost at the 10^th^ culture passage. We hypothesize that this signal loss may be related to the cell senescence and modification of oxidative stress, processes intrinsically linked to melanogenesis [47].

The physical parameters of the measured ESR signal strongly suggested its connection with melanin [17], [20], [48]. Eu- and pheo- melanogenesis occur under low- and high- GSH levels conditions, respectively [49], [50], and changes of GSH levels and increase of oxidative cellular stress are typically observed in melanoma [51].

The presence/absence of ESR signal in different cellular systems may thus depend on factors including senescence level, oxidative stress level, melanogenesis and eu/pheomelanin proportion.

In human primary melanocytes (1.2×10^6^ cells) showing a dark cellular pellet, no ESR signal was recorded (Fig. 1B); on the contrary, melanoma cells SKMEL-28 and SKMEL-110 (1.0×10^6^ cells) show an intense ESR signal (Fig. 1A).

Interestingly, previous data [52] report a clear ESR signal in melanocytes, in apparent contrast with our findings; however it should be noted that such signal was recorded on 100×10^6^ cells, i.e. a number of cells almost hundred times higher as compared to our experimental conditions, indicating that detection of an ESR signal in dark melanocytes strongly depends on the number of cells investigated.

It is well known that melanocytes visual pigmentation consistently correlates with eumelanin but not always with pheomelanin levels [53] and that some melanoma cell lines with high pheomelanin content has not visible pigmentation [54]. Consistently with all such considerations, it is reasonable to speculate that ESR analysis may detect qualitative alteration of the eu/pheomelanin proportion, likely independent form the visual pigmentation and absolute eumelanin content.

The same ESR signal was specifically found in freshly drawn mouse melanoma-tissues as well as in paraffin-embedded primary human melanoma-tissues (Fig. 2) while it was absent in several mouse non-melanoma tissues (Fig. 2B). This signal appeared to be stable for at least 14 days upon freezing, suggesting that the conditions of preservation of the mouse tumours allowed successful measurements repeated in time.

ESR signal recorded in human paraffin-embedded nevi was markedly lower than ESR signal recorded in human melanoma sections (Fig. 2C), further supporting the hypothesis that, under our experimental conditions, endogenous ESR signal may be related to qualitative melanin differences occurring particularly in melanoma. [10], [55]–[58]. The fine analysis of ESR spectra may reveal the specific proportion of eumelanin and pheomelanin (a/b ratio). Comparison of the a/b ratio in melanomas “Low Breslow” and “High Breslow” and in nevi vs “High Breslow” melanomas in the present study indicated a significant difference between the copolymers composition in these patients groups.

It is therefore reasonable to hypothesize that melanin in melanoma cells undergoes a qualitative change associated with deregulation of eumelanin/pheomelanin ratio, leading to paramagnetic melanin-based radicals accumulation [59]. The significant increase of a/b ratio in melanomas “High Breslow” (Fig. 4C) also suggests a contribution of pheomelanin in melanoma [20]; this contribution increases with the progression of melanoma indicating an interesting field of future investigations.

A strong statistical difference of the ESR-signal was measured in human melanoma specimens *versus* nevus samples; this was observed in any subgroup analyzed (such as body location and sex) except in “Limbs” subgroup, indicating that in most cases ESR is consistently and significantly higher in melanomas than in nevi (Fig. 4A).

When all nevi were compared to “Low Breslow” melanomas and “High Breslow” melanomas, ANOVA analysis showed a significant difference as function of Breslow’s depth (Fig. 4B) indicating that ESR analysis may discriminate nevi from melanomas as well as “Low Breslow” from “High Breslow” melanomas, while it is unable to discriminate nevi from melanomas “Low Breslow”. Most interestingly Spearman’s correlation test confirmed such observation, demonstrating a very significant positive correlation between ESR signal and Breslow’s depth, computed with either amplitude and integral values. These observations prompted us to suggest a potential application of ESR-spectroscopy to melanoma diagnosis; such hypothesis was then verified by ROC analysis (Fig. 6), showing a strong and highly significant discriminating ability of ESR signal to identify melanomas from nevi.

ESR technique has been previously suggested for diagnosis and employed in melanoma research [41], [42], however the present study is the first reporting a clear association of a specific ESR signal to a large number (n = 52) of human melanomas using a large number of healthy controls (n = 60 nevi). Furthermore, a different eu/pheomelanin ratio in nevi vs melanomas “High Breslow” has been shown here for the first time, strongly supporting that qualitative melanin changes may occur in nevi as compared to melanomas with worst prognosis.

The quantitative information of ESR spectra is usually expressed in arbitrary units by the integral intensity of the absorption signal. In the present study we report calculations carried out with both amplitudes and double-integrals, which are directly related, provided linewidth is constant. In the measurements performed in the present study no significant variation in linewidth was found for all samples. According to such calculations spectra amplitude was considered a good quantitative approximation [12]. To further support this approximation, correlation of integrals with amplitude was computed in all spectra, giving a very high correlation coefficient (R = 0.89; p<0.0001).

Signal amplitude is the parameter directly measured by the instrument, is easy to be performed by all operators and is more reproducible than the integral calculated value. For these reasons we indicate amplitudes as an effective alternative to integrals, under our experimental conditions.

Although a larger study is needed to further validate this observation in a multicenter study, the present investigation validates the hypothesis that ESR analysis may effectively discriminate human melanomas from human nevi supporting the routine histological diagnostic process.

We believe this study may stimulate further development of skin ESR scanners to open a novel path toward the early non-invasive melanoma diagnosis.

## Supporting Information

Figure S1 Superimposition of the ESR spectra of 8 nevi and 8 melanoma samples randomly taken from the “All Set”. The actual shape of the selected area is reported.
(TIF)

Figure S2 ROC analysis carried on with double integral values. **A)** Nevi vs Melanomas; **B)** Nevi vs Melanomas “Low Breslow”; **C)** Nevi vs Melanomas “High Breslow”; **D)** Melanomas “Low Breslow” vs Melanomas “High Breslow”
(TIF)

## References

[1] Bandarchi B, Ma L, Navab R, Seth A, Rasty G (2010) From melanocyte to metastatic malignant melanoma. Dermatol Res Pract pii: 583748 Epub 2010 Aug 11. 10.1155/2010/583748 PMC2948895 20936153

[2] Spagnolo F, Queirolo P (2012) Upcoming strategies for the treatment of metastatic melanoma. Arch Dermatol Res 304(3): 177–84. 22350184 10.1007/s00403-012-1223-7

[3] Halait H, Demartin K, Shah S, Soviero S, Langland R, et al (2012) Analytical Performance of a Real-time PCR-based Assay for V600 Mutations in the BRAF Gene, Used as the Companion Diagnostic Test for the Novel BRAF Inhibitor Vemurafenib in Metastatic Melanoma. Diagn Mol Pathol 21(1): 1–8. 22306669 10.1097/PDM.0b013e31823b216f

[4] Faraone D, Aguzzi MS, Toietta G, Facchiano AM, Facchiano F, et al (2009) Platelet-derived growth factor-receptor alpha strongly inhibits melanoma growth in vitro and in vivo. Neoplasia 8: 732–42. 10.1593/neo.09408 PMC2713586 19649203

[5] Aguzzi MS, Faraone D, D'Arcangelo D, De Marchis F, Toietta G, et al (2011) The FGF-2-derived peptide FREG inhibits melanoma growth in vitro and in vivo. Mol Ther 19(2): 266–73. 20924364 10.1038/mt.2010.211 PMC3034841

[6] Aguzzi MS, D'Arcangelo D, Giampietri C, Capogrossi MC, Facchiano A (2011) RAM, an RGDS analog, exerts potent anti-melanoma effects in vitro and in vivo. PLoS One 6(10): e25352. 21984914 10.1371/journal.pone.0025352 PMC3184964

[7] Gremel G, Rafferty M, Lau TY, Gallagher WM (2009) Identification and functional validation of therapeutic targets for malignant melanoma. Crit Rev Oncol Hematol 72(3): 194–214. 19328713 10.1016/j.critrevonc.2009.02.004

[8] Argenziano G, Soyer HP (2001) Dermoscopy of pigmented skin lesions–a valuable tool for early diagnosis of melanoma. Lancet Oncol 2(7): 443–9. 11905739 10.1016/s1470-2045(00)00422-8

[9] Matteucci P, Pinder R, Magdum A, Stanley P (2011) Accuracy in skin lesion diagnosis and the exclusion of malignancy. J Plast Reconstr Aesthet Surg 64(11): 1460–5. 21741335 10.1016/j.bjps.2011.06.017

[10] Sarangarajan R, Apte SP (2006) The polymerization of melanin: a poorly understood phenomenon with egregious biological implications. Melanoma Res 16(1): 3–10. 16432450 10.1097/01.cmr.0000195699.35143.df

[11] Riley PA (2003) Melanogenesis and melanoma. Pigment Cell Res 16(5): 548–52. 12950735 10.1034/j.1600-0749.2003.00069.x

[12] Plonka PM (2009) Electron paramagnetic resonance as a unique tool for skin and hair research. Exp Dermatol 18(5): 472–84. 19368555 10.1111/j.1600-0625.2009.00883.x

[13] Slominski A, Tobin DJ, Shibahara S, Wortsman J (2004) Melanin pigmentation in mammalian skin and its hormonal regulation. Physiol Rev 84(4): 1155–228. 15383650 10.1152/physrev.00044.2003

[14] Sarna T, Plonka PM (2005) Biophysical studies of melanin: paramagnetic, ion-exchange and redox properties of melanin pigments and their photoreactivity. In: Eaton SS, Eaton GR, Berliner LJ, eds. Biomedical ESR, Biological Magnetic Resonance Series, vol. 23. The Netherlands-New York- Boston: Kluwer Academic Publishers. 125–146.

[15] Enochs WS, Nilges MJ, Swartz HM (1993) A standardized test for the identification and characterization of melanins using electron paramagnetic resonance (EPR) spectroscopy. Pigment Cell Res 6(2): 91–9. 8391699 10.1111/j.1600-0749.1993.tb00587.x

[16] Blois MS, Zahlan AB, Maling JE (1964) Electron Spin Resonance studies on melanin. Biophys J 4: 471–90. 14232133 10.1016/s0006-3495(64)86797-7 PMC1367600

[17] Commoner B, Townsend J, Pake GE (1954) Free radicals in biological materials. Nature 174(4432): 689–91. 13213980 10.1038/174689a0

[18] Vsevolodov EB, Ito S, Wakamatsu K, Kuchina II, Latypov IF (1991) Comparative analysis of hair melanins by chemical and electron spin resonance methods. Pigment Cell Res 4(1): 30–4. 1656423 10.1111/j.1600-0749.1991.tb00310.x

[19] Meredith P, Sarna T (2006) The physical and chemical properties of eumelanin. Pigment Cell Res 19(6): 572–94. 17083485 10.1111/j.1600-0749.2006.00345.x

[20] Sealy RC, Hyde JS, Felix CC, Menon IA, Prota G (1982) Eumelanins and pheomelanins: characterization by electron spin resonance spectroscopy. Science 217(4559): 545–7. 6283638 10.1126/science.6283638

[21] Nebert DW, Mason HS (1963) An electron spin resonance study of neoplasms. Cancer Res 23: 833–849. 14085278

[22] Slominski A (1983) Rapid melanization of bomirski amelanotic melanoma cells in cell culture. Biosci Rep 3(2): 189–94. 6189530 10.1007/BF01121951

[23] Lund LP, Timmins GS (2007) Melanoma, long wavelength ultraviolet and sunscreens: controversies and potential resolutions. Pharmacol Ther 114(2): 198–207. 17376535 10.1016/j.pharmthera.2007.01.007

[24] Wood SR, Berwick M, Ley RD, Walter RB, Setlow RB, et al (2006) UV causation of melanoma in Xiphophorus is dominated by melanin photosensitized oxidant production. Proc Natl Acad Sci USA 103(11): 4111–5. 16537493 10.1073/pnas.0511248103 PMC1449655

[25] Godechal Q, Gallez B (2011) The contribution of electron paramagnetic resonance to melanoma research. J Skin Cancer 2011: 273280 Epub 2011 Sep 20. 10.1155/2011/273280 PMC3176523 21941659

[26] Godechal Q, Defresne F, Danhier P, Leveque P, Porporato PE, et al (2011) Assessment of melanoma extent and melanoma metastases invasion using electron paramagnetic resonance and bioluminescence imaging. Contrast Media Mol Imaging 6(4): 282–288. 21861288 10.1002/cmmi.430

[27] Cieszka KA, Hill HZ, Hill GJ, Plonka PM (1995) Growth and pigmentation in genetically related S91 Cloudman melanoma cell lines treated with IBMX and MSH. Exp Dermatol 4: 192–198. 8535613 10.1111/j.1600-0625.1995.tb00244.x

[28] Okazaki M, Kuwata K, Miki Y, Shiga S, Shiga T (1985) Electron spin relaxation of synthetic melanin and melanin-containing human tissues as studied by electron spin echo and electron spin resoanance. Arch Biochem Biophys 242: 197–205. 2996430 10.1016/0003-9861(85)90493-x

[29] Elek G, Lapis K, Rockenbauer A (1980) ESR investigation of paraffin-embedded ocular melanomas. Br J Cancer 2: 199–203. 10.1038/bjc.1980.30 PMC2010198 6245671

[30] Pastore S, Potapovich A, Kostyuk V, Mariani V, Lulli D, et al (2009) Plant polyphenols effectively protect HaCaT cells from ultraviolet C-triggered necrosis and suppress inflammatory chemokine expression. Ann N Y Acad Sci 1171: 305–13. 19723070 10.1111/j.1749-6632.2009.04684.x

[31] Giampietri C, Petrungaro S, Coluccia P, D'Alessio A, Starace D, et al (2005) Germ cell apoptosis control during spermatogenesis. Contraception 72(4): 298–302. 16181975 10.1016/j.contraception.2005.04.011

[32] Giampietri C, Petrungaro S, Coluccia P, Antonangeli F, Giannakakis K, et al (2010) c-Flip overexpression affects satellite cell proliferation and promotes skeletal muscle aging. Cell Death Dis 29 1: e38. 10.1038/cddis.2010.17 PMC3032302 21364645

[33] Pedersen JZ, Cox RP (1988) Use of flat glass capillaries as ESR Aqueous sample cells. J Magn Reson 77: 369–371.

[34] Tedeschi AM, Franco L, Ruzzi M, Paduano L, Corvaja C, et al (2003) Micellar aggregation of alkyltrimethylammonium bromide surfactants studied by electron paramagnetic resonance of an anionic nitroxide. Phys Chem Chem Phys 5(19): 4204–4209.

[35] Tarallo R, Accardo A, Falanga A, Guarnieri D, Vitiello G, et al (2011) Clickable functionalization of liposomes with the gH625 peptide from Herpes simplex virus type I for intracellular drug delivery. Chemistry 17(45): 12659–12668. 21956538 10.1002/chem.201101425

[36] Caramuta S, Egyházi S, Rodolfo M, Witten D, Hansson J, et al (2010) MicroRNA expression profiles associated with mutational status and survival in malignant melanoma. J Invest Dermatol 130(8): 2062–70. 20357817 10.1038/jid.2010.63

[37] Prasmickaite L, Engesaeter BØ, Skrbo N, Hellenes T, Kristian A, et al (2010) Aldehyde dehydrogenase (ALDH) activity does not select for cells with enhanced aggressive properties in malignant melanoma. PLoS One 5(5): e10731. 20505780 10.1371/journal.pone.0010731 PMC2874003

[38] Kalbasi A, Fonsatti E, Natali PG, Altomonte M, Bertocci E, et al (2010) CD40 expression by human melanocytic lesions and melanoma cell lines and direct CD40 targeting with the therapeutic anti-CD40 antibody CP-870,893. J Immunother 33(8): 810–6. 20842056 10.1097/CJI.0b013e3181ee73a7

[39] Berliner LJ, Fujii H, Wan XM, Lukiewicz SJ (1987) Feasibility study of imaging a living murine tumor by electron paramagnetic resonance. Magn Reson Med 4(4): 380–4. 3035320 10.1002/mrm.1910040410

[40] Katsuda H, Kobayashi T, Saito H, Matsunaga T, Ikeya M (1990) Electron spin resonance imaging of mouse B16 melanoma. Chem Pharm Bull (Tokyo) 38(10): 2838–40. 1963815 10.1248/cpb.38.2838

[41] Godechal Q, Leveque P, Marot L, Baurain JF, Gallez B (2012) Optimization of electron paramagnetic resonance imaging for visualization of human skin melanoma in various stages of invasion. Exp Dermatol 21(5): 341–6. 22509830 10.1111/j.1600-0625.2012.01461.x

[42] Vanea E, Charlier N, Dewever J, Dinguizli M, Feron O, et al (2008) Molecular electron paramagnetic resonance imaging of melanin in melanomas: a proof-of-concept. NMR Biomed 21(3): 296–300. 18246539 10.1002/nbm.1241

[43] Farnaud S, Amini M, Rapisarda C, Cammack R, Bui T, et al (2008) Biochemical and spectroscopic studies of human melanotransferrin (MTf): electron-paramagnetic resonance evidence for a difference between the iron-binding site of MTf and other transferrins. Int J Biochem Cell Biol 40(12): 2739–45. 18691669 10.1016/j.biocel.2008.07.003

[44] Haywood R, Rogge F, Lee M (2008) Protein, lipid, and DNA radicals to measure skin UVA damage and modulation by melanin. Free Radic Biol Med 44(6): 990–1000. 18160051 10.1016/j.freeradbiomed.2007.11.019

[45] Dikalov S, Losik T, Arbiser JL (2008) Honokiol is a potent scavenger of superoxide and peroxyl radicals. Biochem Pharmacol 76(5): 589–96. 18640101 10.1016/j.bcp.2008.06.012 PMC2575413

[46] Creasey AA, Smith HS, Hackett AJ, Fukuyama K, Epstein WL, et al (1979) Biological properties of human melanoma cells in culture. In Vitro 15(5): 342–50. 478563 10.1007/BF02616140

[47] Galván I, Alonso-Alvarez C, Negro JJ (2012) Relationships between Hair Melanization, Glutathione Levels, and Senescence in Wild Boars. Physiol Biochem Zool 85(4): 332–347. 22705484 10.1086/666606

[48] Mason HS, Ingram DJ, Allen B (1960) The free radical property of melanins. Arch Biochem Biophys 86: 225–30. 14422053 10.1016/0003-9861(60)90409-4

[49] Benathan M, Virador V, Furumura M, Kobayashi N, Panizzon RG, et al (1999) Co-regulation of melanin precursors and tyrosinase in human pigment cells: roles of cysteine and glutathione. Cell Mol Biol (Noisy-le-grand) 45(7): 981–90. 10644002

[50] Galván I, Solano F (2009) The evolution of eu- and pheomelanic traits may respond to an economy of pigments related to environmental oxidative stress. Pigment Cell Melanoma Res 22(3): 339–42. 19243532 10.1111/j.1755-148X.2009.00559.x

[51] Wittgen HG, van Kempen LC (2007) Reactive oxygen species in melanoma and its therapeutic implications. Melanoma Res 17(6): 400–9. 17992124 10.1097/CMR.0b013e3282f1d312

[52] Hill HZ, Hill GJ, Cieszka K, Plonka PM, Mitchell DL, et al (1997) Comparative action spectrum for ultraviolet light killing of mouse melanocytes from different genetic coat color backgrounds. Photochem Photobiol 65(6): 983–9. 9188277 10.1111/j.1751-1097.1997.tb07958.x

[53] Wakamatsu K, Kavanagh R, Kadekaro AL, Terzieva S, Sturm RA, et al (2006) Diversity of pigmentation in cultured human melanocytes is due to differences in the type as well as quantity of melanin. Pigment Cell Res 19(2): 154–62. 16524431 10.1111/j.1600-0749.2006.00293.x

[54] del Marmol V, Ito S, Jackson I, Vachtenheim J, Berr P, et al (1993) TRP-1 expression correlates with eumelanogenesis in human pigment cells in culture. FEBS Lett 327(3): 307–10. 8348959 10.1016/0014-5793(93)81010-w

[55] Bonnet M, Mishellany F, Papon J, Cayre A, Penault-Llorca F, et al (2010) Anti-melanoma efficacy of internal radionuclide therapy in relation with melanin target distribution. Pigment Cell Melanoma Res 23(5): e1–11. 10.1111/j.1755-148X.2010.00716.x 20444199

[56] Ren G, Miao Z, Liu H, Jiang L, Limpa-Amara N, et al (2009) Melanin-targeted preclinical PET imaging of melanoma metastasis. J Nucl Med 50(10): 1692–9. 19759116 10.2967/jnumed.109.066175 PMC4215196

[57] Eichhorn R, Wessler G, Scholz M, Leupold D, Stankovic G, et al (2009) Early diagnosis of melanotic melanoma based on laser-induced melanin fluorescence. J Biomed 14(3): 034033. 10.1117/1.3155511 19566326

[58] Dimitrow E, Riemann I, Ehlers A, Koehler MJ, Norgauer J, et al (2009) Spectral fluorescence lifetime detection and selective melanin imaging by multiphoton laser tomography for melanoma diagnosis. Exp Dermatol 18(6): 509–15. 19243426 10.1111/j.1600-0625.2008.00815.x

[59] Latocha M, Pilawa B, Chodurek E, Buszman E, Wilczok T (2004) Paramagnetic centers in tumor cells. Appl Magn Reson (26): 339–344.

